# Supplementary material for: Causal contribution of optic flow signal in Macaque extrastriate visual cortex for roll perception
Source: Nat Commun. 2022 Sep 19;13:5479. doi: 10.1038/s41467-022-33245-5 (PMC9485245; doi:10.1038/s41467-022-33245-5)
Supplement: Supplementary file 1 — Supplementary Information [file 41467_2022_33245_MOESM1_ESM.pdf]

Supplementary Information for "Causal Contribution of Optic Flow Signal in Macaque Extrastriate Visual Cortex for Roll Perception"

Li et al.

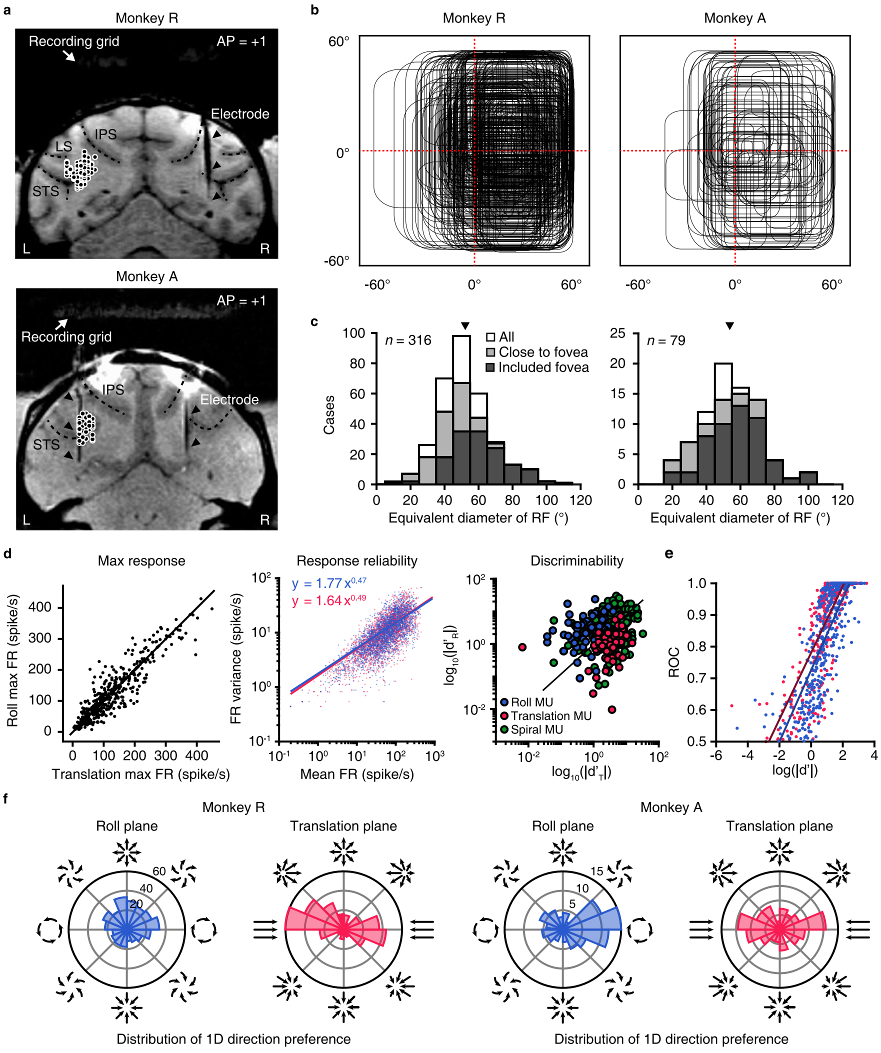

Supplementary Figure 1. Locations and response properties of recording sites in MSTd. (**a**) Magnetic resonance imaging (MRI) reconstruction of recording sites in the MSTd for monkey R (top) and monkey A (bottom). Notice that all recording session along the anterior-posterior (AP) axis were projected onto a single coronal plane (AP +1). (**b**) Size, location and (**c**) distribution of receptive field (RF) of MSTd MUs. Dark bars, RFs included the fovea; Grey bars, RFs came close to the fovea but not included (proximal edge $\leqslant$ 3° from the fixation point); Open bars, the rest. (**d**) Comparison of response to roll and translation stimuli from maximum response (Pearson's correlation coefficient = 0.93, p = 3.4e-191; black line, linear regression fit, left panel), response reliability (response variance and average response computed across trials, power functions were fit to the data for roll and translation conditions. middle panel), and direction discriminability (Pearson' correlation coefficient = 0.28, p = 1.4e-9; black line, linear regression fit, right panel). (**e**) Comparison of neural discriminability defined by d' and ROC analysis. Each dot represents one recording sites. Roll plane (red), r = 0.79, p = 6.9e-97; Translation plane (blue), r = 0.82, p = 1.3e-105, Pearson's correlation. Colored line, linear regression fit. (**f**) 1D direction preference for roll plane (red) and translation plane (blue) across animals.

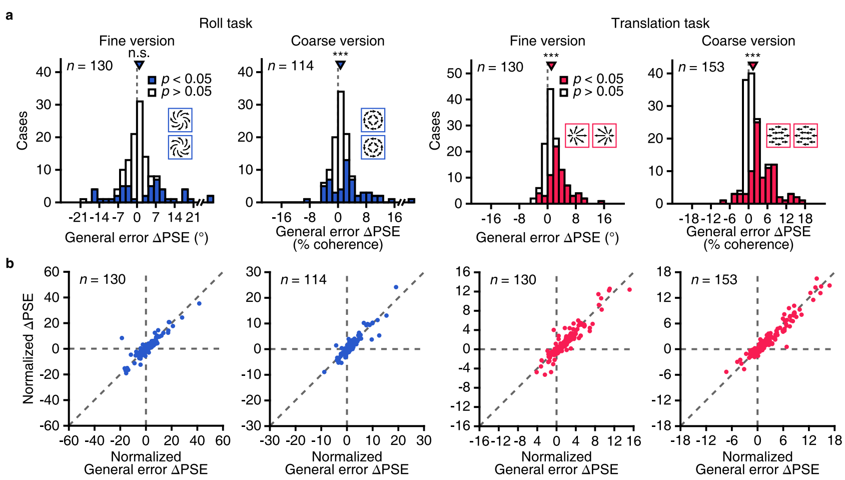

Supplementary Figure 2. Microstimulation induced general error PSE shift including all trials within each flow pattern. (**a**) Distributions of general error psychometric functions shift at subjective quarter point for fine and coarse versions. Fine-roll, median ΔPSE = 0.84°, p = 0.14; coarse-roll, median ΔPSE = 0.7%, p = 2.6e-4; fine-translation, median ΔPSE = 1.1°, p = 4.2e-9; coarse-translation, median ΔPSE = 1.4%, p = 1.3e-11 (two-tail sign test). Same format as Fig. 4b. (**b**) Comparison of ΔPSE calculated from trials with correct flow pattern choice and trials with wrong inter-flow pattern choice. Fine-roll, r = 0.90, p = 1.5e-48; coarse-roll, r = 0.93, p = 8.7e-50; fine-translation, r = 0.93, p = 7e-57; coarse-translation, r = 0.97, p = 6.6e-90 (two-tail Pearson's correlation).

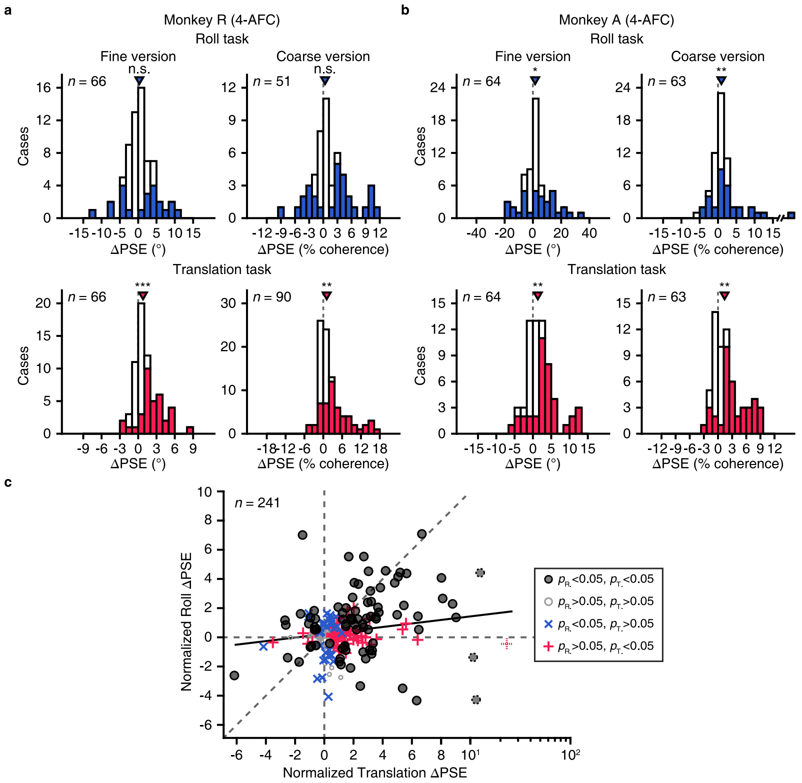

Supplementary Figure 3. Summary of microstimulation-induced PSE shifts across paradigms and animals. (**a**, **b**) Summary of ΔPSE for two animals. Monkey R: fine-roll, median ΔPSE = 0.54°, $p = 0.32$; coarse-roll, median ΔPSE = 0.37%, $p = 0.092$; fine-translation, median ΔPSE = 0.79°, $p = 6.4\text{e-}6$; coarse-translation, median ΔPSE = 1.1%, $p = 2.1\text{e-}3$; Monkey A: fine-roll, median ΔPSE = 1.6°, $p = 0.033$; coarse-roll, median ΔPSE = 0.93%, $p = 5.2\text{e-}3$; fine-translation, median ΔPSE = 1.3°, $p = 3.7\text{e-}3$; coarse-translation, median ΔPSE = 1.4%, $p = 5.2\text{e-}3$ (two-tail sign test). Same format as Fig. 4b. (**c**) Comparison of ΔPSE between roll task and translation task. Filled circles, sites with significant ΔPSE under both roll and translation task; Crosses, sites with significant ΔPSE under either one task; Open circles, sites with nonsignificant ΔPSE under both task. Two-tail Pearson's correlation coefficient = 0.21, $p = 1.4\text{e-}3$. Black line, linear regression fit. Dotted symbols, x-axis in log scale.

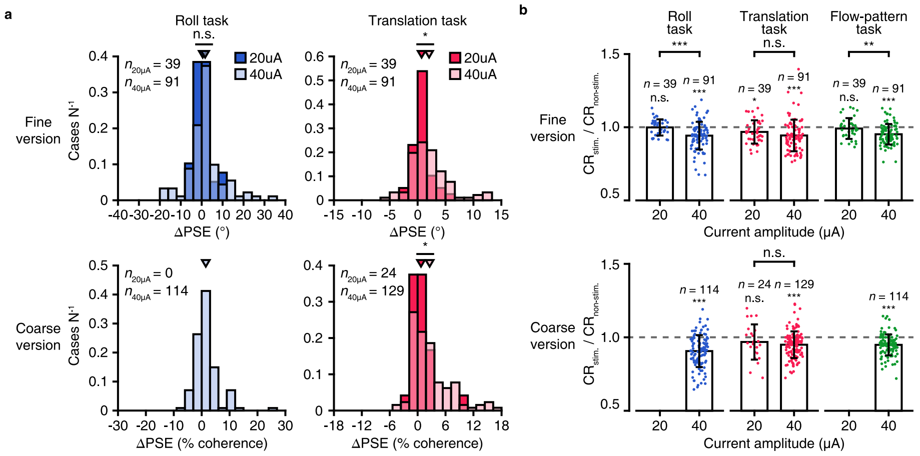

Supplementary Figure 4. Comparison of microstimulation effect across two current amplitudes. (**a**) Microstimulation induced PSE shift for 20 μA (dark colored) and 40 μA (light colored) electrical current. Asterisks, significance of the difference between median ΔPSE stimulated by 20 μA and 40 μA (n.s., not significant; *$p < 0.05$, two-tail t-test). Fine-roll, $p = 0.42$; fine-translation, $p = 0.026$; coarse-translation, $p = 0.034$. (**b**) Microstimulation induced correct rate (CR) change for 20 μA and 40 μA. Asterisk above dots, mean ΔPSE significantly different from zero (two-tail t-test). For 20 μA: fine-roll, $p = 0.89$; fine-translation, $p = 0.016$; fine-flow-pattern, $p = 0.46$; coarse-translation, $p = 0.21$; For 40 μA: fine-roll, $p = 9.8e\text{-}8$; fine-translation, $p = 3.0e\text{-}6$; fine-flow-pattern, $p = 4.3e\text{-}9$; coarse-roll, $p = 2.2e\text{-}15$; coarse-translation, $p = 4.2e\text{-}9$; coarse-flow-pattern, $p = 6.7e\text{-}12$. Error bar, STD. Horizontal lines, significance of the difference between CR change simulated by 20 μA and 40 μA (n.s., not significant; **$p < 0.01$; ***$p < 0.001$, two-tail non-paired t-test). Fine-roll, $p = 7.2e\text{-}4$; fine-translation, $p = 0.21$; fine-flow-pattern, $p = 4.2e\text{-}3$; coarse-translation, $p = 0.38$.

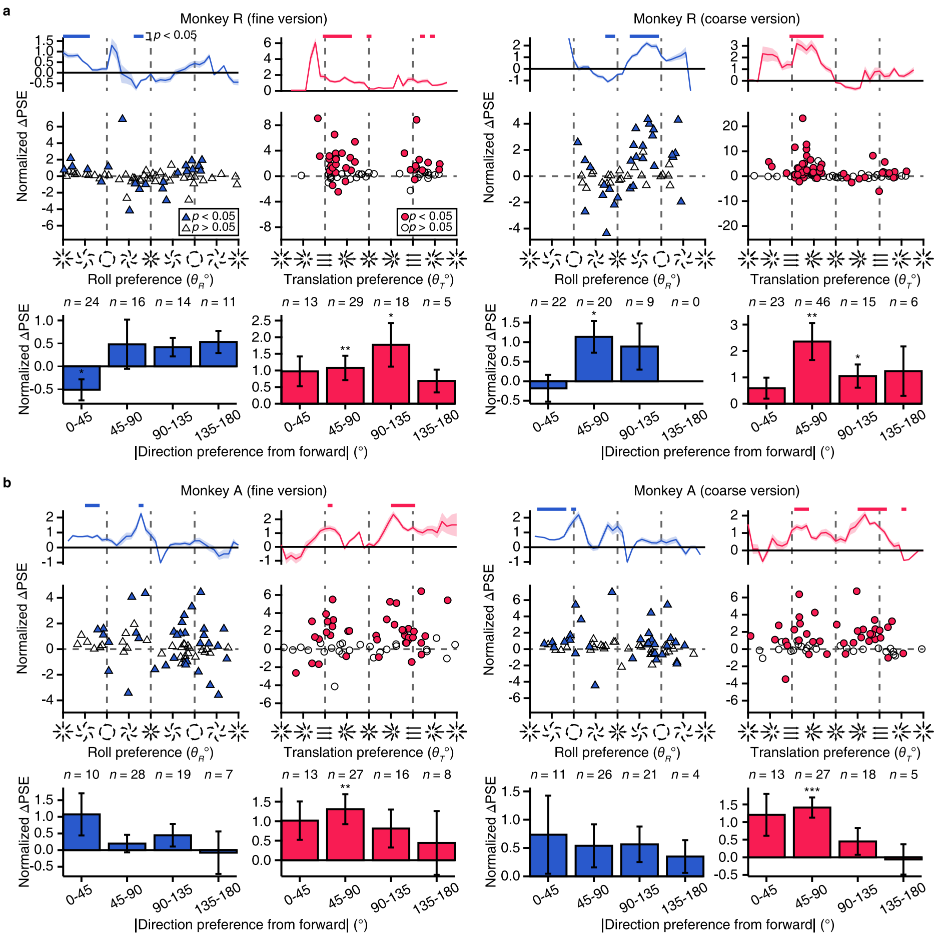

Supplementary Figure 5. Direction preference modulation of microstimulation effect on PSE shift across task versions and animals. Relationship between ΔPSE and direction preference for monkey R (**a**) and monkey A (**b**) under fine (left two columns) and coarse (right two columns) version. Same format as Fig. 5a. Error bar, SEM.

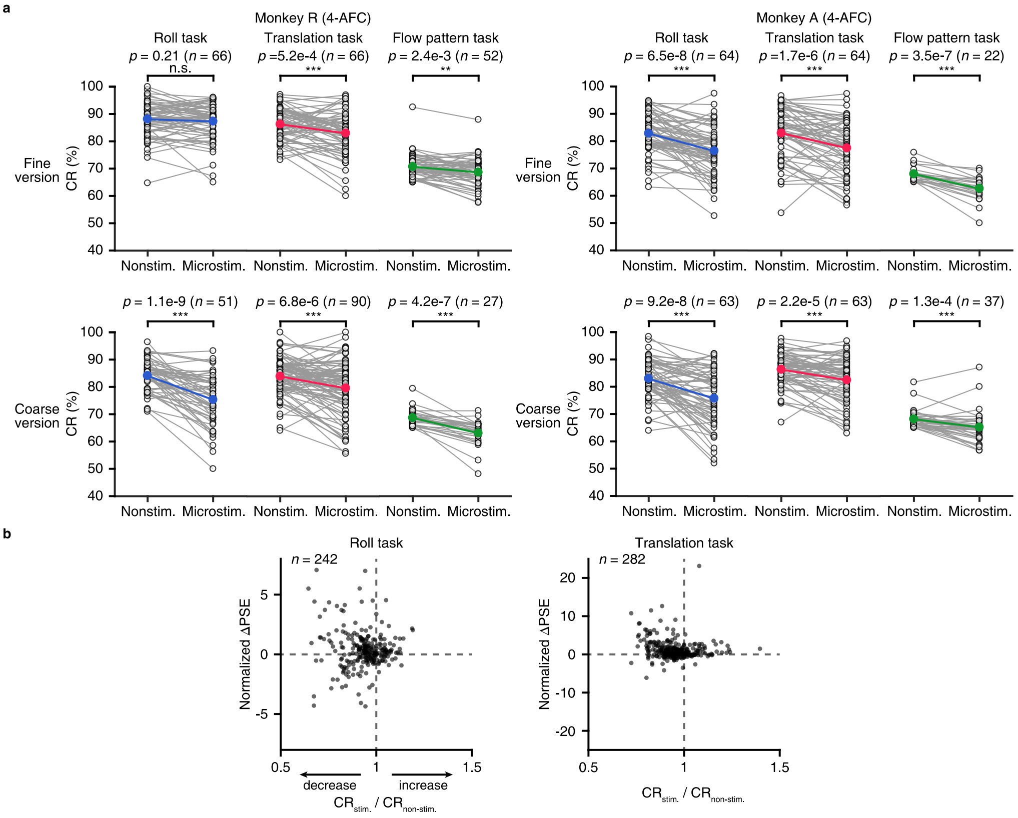

Supplementary Figure 6. Microstimulation effect on the animals' performance discriminability across animals. (**a**) Microstimulation-induced change of CR under fine and coarse version across animals. Horizontal lines, *p* value for two-tail paired t-test. n.s. not significant; $^{**}p < 0.01$; $^{***}p < 0.001$. Monkey R: fine-roll, 0.93% in CR reduction ($p = 0.21$, the same hereinafter); fine-translation, 3.8% ($p =$ 5.2e-4); fine-flow-pattern, 2.9% ($p =$ 2.4e-3); coarse-roll, 10% ($p =$ 1.1e-9); coarse-translation, 5.2% (p = 6.8e-6); coarse-flow-pattern, 8.1% ($p =$ 4.2e-7). Monkey A: fine-roll, 7.7% in CR reduction ($p =$ 6.5e-8); fine-translation, 6.6% ($p =$ 1.7e-6); fine-flow-pattern, 7.9% ($p =$ 3.5e-7); coarse-roll, 8.7% ($p =$ 9.2e-8); coarse-translation, 4.5% ($p =$ 2.2e-5); coarse-flow-pattern, 4.4% ($p =$ 1.3e-4). Same format as Fig. 7b. (**b**) Scatter plot of normalized ΔPSE as a function of induced CR change.

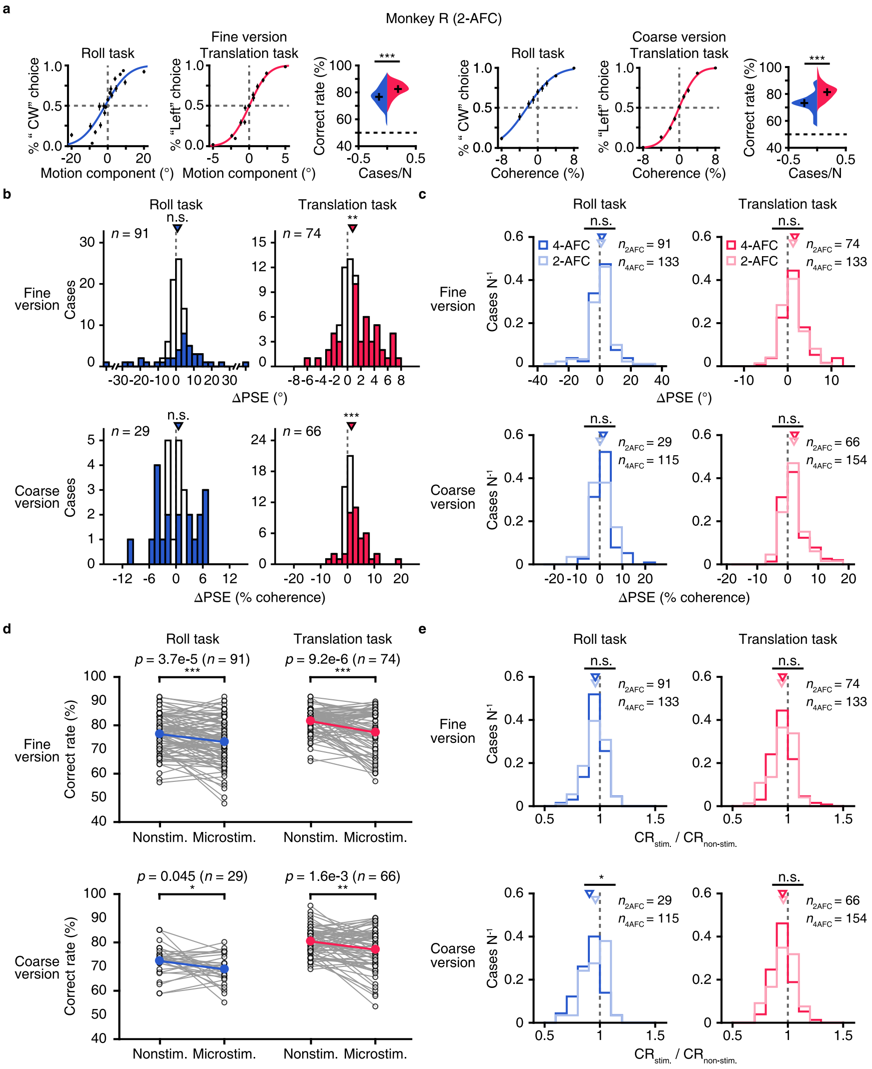

Supplementary Figure 7. Microstimulation effect in 2-AFC task. (**a**) Average psychometric functions for roll task (blue) and translation task (red) under fine and coarse versions. In fine-roll paradigm, n = 91, μ = -1.5°, σ = 10.2°; in fine-translation paradigm, n = 74, μ = 0.014°, σ = 2.1°; in coarse-roll paradigm, n = 29, μ = -2.4%, σ = 4.8%; in coarse-translation paradigm, n = 66, μ = -0.012%, σ = 3.0%. Error bar, SEM. Histograms show distributions of the CR for roll task (blue) and translation task (red) under fine and coarse versions. Fine version, p = 5.6e-6; Coarse version, p = 1.9e-7. Dashed line, 50% guess rate. Same format as Fig. 2. (**b**) Summary of microstimulation-induced PSE shifts in 2-AFC task for fine and coarse versions. fine-roll, median ΔPSE = 0.83°, p = 0.059; coarse-roll, median ΔPSE = 0.55%, p = 0.71; fine-translation, median ΔPSE = 0.77°, p = 7.1e-3; coarse-translation, median ΔPSE = 1.5%, p = 7.6e-4 (two-tail sign test). Same format as Fig. 4b. (**c**) Comparison of microstimulation induced PSE shift between 2-AFC task (light colored) and 4-AFC task (dark colored). Horizontal lines, significance of the difference between 2-AFC task and 4-AFC task (n.s., not significant, two-tail non-paired t-test). (**d**) Summary of microstimulation-induced CR change in 2-AFC task for fine and coarse versions. Horizontal lines, p value for two-tail paired t-test. *$p < 0.05$, **$p < 0.01$, ***$p < 0.001$. Fine-roll, 4.2% in CR reduction (p = 3.7e-5, the same hereinafter); fine-translation, 5.7% (p = 9.2e-6); coarse-roll, 4.7% (p = 0.045); coarse-translation, 4.2% (p = 1.6e-3). Same format as Fig. 7b-c. (**e**) Comparison of microstimulation induced CR change between 2-AFC task (light colored) and 4-AFC task (dark colored). Horizontal lines, significance of the difference between 2-AFC task and 4-AFC task (n.s., not significant;

*$p<0.05$, two-tail non-paired t-test). Fine-roll, $p = 0.87$; fine-translation, $p = 0.59$; coarse-roll, $p = 0.018$; coarse-translation, $p = 0.91$.
