# Supplementary material for: Causal contribution of optic flow signal in Macaque extrastriate visual cortex for roll perception
Source: Nat Commun. 2022 Sep 19;13:5479. doi: 10.1038/s41467-022-33245-5 (PMC9485245; doi:10.1038/s41467-022-33245-5)
Supplement: Supplementary file 3 — Reporting Summary [file 41467_2022_33245_MOESM3_ESM.pdf]

nature portfolio

Corresponding author(s): *Double-blind peer review submissions: write DBPR and your manuscript number here instead of author names.*

Last updated by author(s): Aug 9, 2022

Reporting Summary

Nature Portfolio wishes to improve the reproducibility of the work that we publish. This form provides structure for consistency and transparency in reporting. For further information on Nature Portfolio policies, see our Editorial Policies and the Editorial Policy Checklist.

Statistics

For all statistical analyses, confirm that the following items are present in the figure legend, table legend, main text, or Methods section.

n/a	Confirmed	
☐	☒	The exact sample size (*n*) for each experimental group/condition, given as a discrete number and unit of measurement
☐	☒	A statement on whether measurements were taken from distinct samples or whether the same sample was measured repeatedly
☐	☒	The statistical test(s) used AND whether they are one- or two-sided<br>*Only common tests should be described solely by name; describe more complex techniques in the Methods section.*
☐	☒	A description of all covariates tested
☒	☐	A description of any assumptions or corrections, such as tests of normality and adjustment for multiple comparisons
☐	☒	A full description of the statistical parameters including central tendency (e.g. means) or other basic estimates (e.g. regression coefficient) AND variation (e.g. standard deviation) or associated estimates of uncertainty (e.g. confidence intervals)
☐	☒	For null hypothesis testing, the test statistic (e.g. *F*, *t*, *r*) with confidence intervals, effect sizes, degrees of freedom and *P* value noted<br>*Give P values as exact values whenever suitable.*
☒	☐	For Bayesian analysis, information on the choice of priors and Markov chain Monte Carlo settings
☒	☐	For hierarchical and complex designs, identification of the appropriate level for tests and full reporting of outcomes
☐	☒	Estimates of effect sizes (e.g. Cohen's *d*, Pearson's *r*), indicating how they were calculated

Our web collection on statistics for biologists contains articles on many of the points above.

Software and code

Policy information about availability of computer code

Data collection	A custom designed data acquisition system was used for neural recordings, electrical microstimulation, and for aligning spikes with stimulus and behavioral data.
Data analysis	MATLAB R2017a functions were used to analyze all data.

For manuscripts utilizing custom algorithms or software that are central to the research but not yet described in published literature, software must be made available to editors and reviewers. We strongly encourage code deposition in a community repository (e.g. GitHub). See the Nature Portfolio guidelines for submitting code & software for further information.

Data

Policy information about availability of data

All manuscripts must include a data availability statement. This statement should provide the following information, where applicable:

- Accession codes, unique identifiers, or web links for publicly available datasets
- A description of any restrictions on data availability
- For clinical datasets or third party data, please ensure that the statement adheres to our policy

Source Data are provided with this paper for all data presented in graphs within the figures. The raw data of behavioral and neurophysiology in this study have been deposited in the Zenodo database and are accessible at https://doi.org/10.5281/zenodo.6956565.

Field-specific reporting

Please select the one below that is the best fit for your research. If you are not sure, read the appropriate sections before making your selection.

☒ Life sciences ☐ Behavioural & social sciences ☐ Ecological, evolutionary & environmental sciences

For a reference copy of the document with all sections, see nature.com/documents/nr-reporting-summary-flat.pdf

Life sciences study design

All studies must disclose on these points even when the disclosure is negative.

Sample size	533 multi-unit (MU, n=444 and 89 for monkey R and monkey A, respectively) recorded from primate MSTd for tuning measurement. Among them, 130 MU used in 4AFC fine roll task, 114 MU used in 4AFC coarse roll task, 130 MU used in 4AFC fine translation task, 153 MU used in 4AFC coarse translation task, 91 MU used in 2AFC fine roll task, 29 MU used in 2AFC coarse roll task, 74 MU used in 2AFC fine translation task, 66 MU used in 2AFC coarse translation task. All 2AFC task data from monkey R. One same MU can be used for more than one task.
Data exclusions	Untuned neurons (n=85 and 4 for monkey R and monkey A, respectively) were not included in behavioral task.
Replication	We replicated the analysis performed on monkey R and monkey A. As described in the manuscript, all main results were replicated in both monkeys.
Randomization	Visual stimulus, stimulated trials and non-stimulated trials, roll and translation condition were randomized on a trial-by-trial basis. Fine and coarse version, 4AFC and 2AFC paradigm were randomized on a block-by-block basis.
Blinding	Blinding is not relevant to this study.

Reporting for specific materials, systems and methods

We require information from authors about some types of materials, experimental systems and methods used in many studies. Here, indicate whether each material, system or method listed is relevant to your study. If you are not sure if a list item applies to your research, read the appropriate section before selecting a response.

Materials & experimental systems

n/a	Involved in the study	
☒	☐	Antibodies
☒	☐	Eukaryotic cell lines
☒	☐	Palaeontology and archaeology
☐	☒	Animals and other organisms
☒	☐	Human research participants
☒	☐	Clinical data
☒	☐	Dual use research of concern

Methods

n/a	Involved in the study	
☒	☐	ChIP-seq
☒	☐	Flow cytometry
☒	☐	MRI-based neuroimaging

Animals and other organisms

Policy information about studies involving animals; ARRIVE guidelines recommended for reporting animal research

Laboratory animals	two male rhesus monkeys (weighing 9~10 kg)
Wild animals	N/A
Field-collected samples	N/A
Ethics oversight	All animal procedures were approved by the Animal Care Committee of Shanghai Institutes for Biological Science, Chinese Academy of Sciences.

Note that full information on the approval of the study protocol must also be provided in the manuscript.
